# Segmentation of White Blood Cells through Nucleus Mark Watershed Operations and Mean Shift Clustering

**DOI:** 10.3390/s150922561

**Published:** 2015-09-08

**Authors:** Zhi Liu, Jing Liu, Xiaoyan Xiao, Hui Yuan, Xiaomei Li, Jun Chang, Chengyun Zheng

**Affiliations:** 1 School of Information Science and Engineering, Shandong University, Jinan 250100, China; E-Mails: liuzhi@sdu.edu.cn (Z.L.); youxiang7799@126.com (J.L.); lz505@163.com (H.Y.); changjun@sdu.edu.cn (J.C.); 2 Department of Nephropathy, Qilu Hospital of Shandong University, Jinan 250012, China; E-Mail: xiaoyanxiao2007@163.com; 3 Department of Oncology, the Second Hospital of Shandong University, Jinan 250033, China; E-Mail: sdulixiaomei@163.com; 4 Department of Hematology, the Second Hospital of Shandong University, Jinan 250033, China

**Keywords:** segment, white blood cells, peripheral blood and bone marrow, different lights, mean shift clustering, C channel component, nucleus mark watershed operation, morphological operations

## Abstract

This paper presents a novel method for segmentation of white blood cells (WBCs) in peripheral blood and bone marrow images under different lights through mean shift clustering, color space conversion and nucleus mark watershed operation (NMWO). The proposed method focuses on obtaining seed points. First, color space transformation and image enhancement techniques are used to obtain nucleus groups as inside seeds. Second, mean shift clustering, selection of the C channel component in the CMYK model, and illumination intensity adjustment are employed to acquire WBCs as outside seeds. Third, the seeds and NMWO are employed to precisely determine WBCs and solve the cell adhesion problem. Morphological operations are further used to improve segmentation accuracy. Experimental results demonstrate that the algorithm exhibits higher segmentation accuracy and robustness compared with traditional methods.

## 1. Introduction

White blood cells (WBCs) in peripheral blood and bone marrow play a significant role in the auxiliary diagnosis of various diseases, such as AIDS, leukemia, and other blood-related diseases. The WBC count, also known as the differential blood count (DBC), is an indicator of certain diseases. In DBC, medical experts count 100 or 200 WBCs on slides stained with blood and accordingly compute the percentage occurrence of each type of WBCs [1]. Traditional counting methods that involve the use of a microscope are time consuming, complicated, tedious, and prone to errors. Meanwhile, automatic recognition methods utilize a flow cytometry apparatus [2] and a blood cell analyzer [3]. These tools are mainly employed for routine blood examination rather than blood cell detection. However, experts always employ blood smears from patients and a microscope to observe the shape of blood cells for the clinical diagnosis of blood diseases in patients. As such, development of an automatic cell recognition system based on image processing and pattern recognition technology to replace manual recognition and counting has been the current trend.

Blood contains different cell lines, the most important of which are the WBCs, platelet, and red blood cells (RBCs) [4]. WBCs, which are also called immune cells, can help the body to fight infection and external matter. Inflammation in the body or other blood diseases can cause changes in the percentage and total numbers of WBC. Collected image samples contain both WBCs and RBCs, thereby influencing the processing and selection of WBCs. In this regard, the WBC segmentation algorithm should accurately work on both peripheral blood and leukemic cells in image processing for precise diagnosis of blood diseases.

Several approaches have been developed for WBC segmentation. These methods are usually based on color space and mathematical morphology operations. Putzu *et al.* [5,6] proposed a method based on the cyan, magenta, yellow, and key plate (CMYK) color space to separate WBCs because these cells lack the Y component. This method solved the problem of distinguishing similarities between the cytoplasm and the background. Putzu *et al.* [5] also used the component in the CIE Lab color space to obtain the nucleus in a leukemic blood image. The hue, saturation, and intensity (HSI) model [7,8,9] is commonly used in numerous color models. Dividing the nucleus only in a peripheral blood image is easy and rapid and could yield improved segmentation accuracy. Lim *et al.* [9] employed a combination of the HSI color space, watershed technique [10,11], and other morphology operations to obtain WBCs in leukocytes and blast cells. The nucleus of WBCs has been segmented using techniques that allow contrast enhancement on grayscale images for noise elimination [12]. These methods are simple but incapable for accurately segmenting the nucleus of WBCs when the gray value of the nucleus is close to the cytoplasm in acute myelocytic leukemia (AML) blood images.

Several researchers have combined the CIE Lab color space with the K-means clustering algorithm for cell division [13,14,15]. The K-means is an algorithm based on color pixel values, with the Euclidean distance as the similarity measure. The algorithm relies excessively on initialization data and other parameters. The clustering result is closely related to the number and shape of the target data. When the color of the cytoplasm strikingly differs from that of the nucleus, this strategy cannot precisely obtain all WBCs in several image conditions.

Rezatofighi *et al.* [16] segmented the nucleus of five types of WBCs in peripheral blood via a novel method based on Gram-Schmidt orthogonalization to amplify the desired color vectors and weaken undesired ones; in this method, the nucleus boundary was employed as the initial contour of the snake to track the boundaries of WBCs. Ko *et al.* [17] proposed a WBC segmentation algorithm with stepwise merging rules through mean shift clustering and boundary removal with a gradient vector flow snake. These algorithms are highly effective and accurate in distinguishing cells. However, these approaches require a significant amount of time and cannot address the problem of overlapping WBCs.

Previous studies mainly used active contour models [18,19,20] and mathematical morphology [10,11,21,22,23] to segment overlapping WBCs. However, excessive segmentation, low accuracy of cell division, and other challenges remain and must be overcome.

The present study mainly aims to develop a new algorithm based on color space, mean shift clustering, illumination adjustment, and nucleus mark watershed operation (NMWO) for segmentation of overlapping WBCs in peripheral blood and AML blood images. Morphological operations such as are morphological reconstruction, open-and-close operations, morphological denoising, and cell centroid connection are employed to improve segmentation accuracy.

The remainder of the paper is organized as follows: Section 2 presents a brief introduction to the theories of color space, mean shift clustering, and watershed transform. Section 3 introduces the proposed framework for WBC segmentation in five stages. Section 4 depicts and discusses the experimental results. Finally, Section 5 summarizes the conclusions of this study.

## 2. Technological Background

Segmentation of WBCs is the most crucial step in hematological image analysis. In clinical blood analysis, medical experts generally use the color features and morphology information of blood cells to distinguish cell types. The proposed method employs different color spaces to identify WBCs, as well as mean shift clustering, C channel component selection, illumination intensity adjustment and image enhancement techniques to obtain a complete set of WBCs. Watershed transformation is also applied to address cell adhesion problem.

### 2.1. Different Color Spaces

The original stained blood smear image is represented by the RGB color space in the RGB model. An RGB image consists of color pixels of M × N × 3 array. Each pixel in a specific spatial location corresponds to red (R), green (G), and blue (B), which are the three components and primary colors for the superposition of different color levels to produce different colors. Thus far, the RGB color space is one of the most widely used color systems.

The HSI color model consists of three components, hue (H), saturation (S), and intensity (I). This model has a color description consistent with that of humans. The HSI space is more appropriate than the RGB space for WBC segmentation because of its low correlation with image processing.

The CMYK color model is a subtractive model used in color printing and describing the printing process. CMYK refers to the four inks used in color printing; C denotes cyan, M refers to magenta, Y denotes yellow, and K depicts the key plate (black) [6].

### 2.2. Illumination Intensity Adjustment

In practice, color information and morphological information are used for WBC recognition. Color information, plays an important role in the image segmentation. The different illumination can produce dissimilarities in the image color. However, illumination is difficult to be standardized in different labs. Therefore, the effect of light intensity should be eliminated during image segmentation.

The methods of color feature extraction mainly include color histogram, chromaticity histogram, and color constancy [24]. A chromaticity histogram not only has the advantages of a color histogram but also can eliminate the influence of light intensity when changing image color. Color constancy can eliminate the influence of light intensity and demonstrate robustness in illumination of color changes, however the corresponding calculation procedure is complex. In this paper, the proposed method transforms the image from the RGB space to the red-green (rg) chroma space to eliminate the influence of illumination intensity.

**Figure 1:**
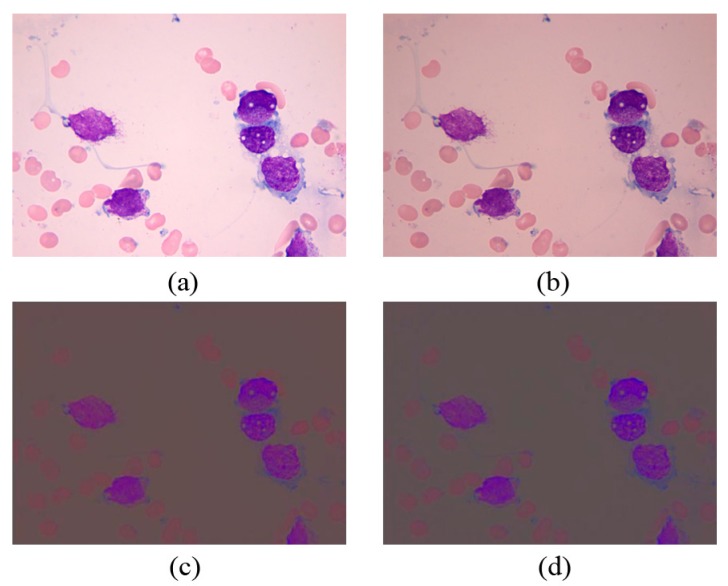
(**a**,**b**) Original RGB image under different light conditions; (**c**) Rg chroma space under light condition of (a); (**d**) Rg chroma space under light condition of (**b**).

The rg chroma space can eliminate the influence of light intensity on color. The color space conversion from the RGB space to the rg chroma space is shown in the following equation:

(1) $$\left\{ \begin{array}{l} r=\frac{R}{R+G+B}\\ g=\frac{G}{R+G+B}\\ b=1-r-g \end{array}\right.$$

where R,G,B shows the three channel components of the original RGB image; r,g,b shows the three channel components in the rg chroma space. Figure 1a shows the original RGB image under different light conditions, whereas Figure 1b shows the same image in the rg chroma space.

### 2.3. Mean Shift Clustering

Mean shift clustering is a gradient ascent method used to determine the local highest density of a data set by using mean shifts. Although the procedure was initially described decades ago [25], it was unpopular in the vision community until its potential uses for feature space analysis and optimization were understood [26,27,28]. The non-parametric nature of mean shift makes this method an effective tool to discover arbitrarily shaped clusters in the data.

Based on the assumption that *n* sample points in *x_i_*, where i=1,2,…,n , are given in the d -dimensional space R^d^, the basic form of the *x* mean shift vector is defined as:

(2) $$M_{h}\left(x\right)=\frac{1}{k}\sum_{x_{i}\in s_{h}}\left(x_{i}-x\right)$$

where *h* is the radius, and *S_h_* is the radius of the high-dimensional spherical area, which satisfies the following relationship of the *y* point set:

(3) $$S_{h}(x)=\{y:{\left(y-x\right)}^{T}(y-x)\leq h^{2}\}$$

where *k* implies that *k* points exist in *x_i_* and falls in the area of *S_h_*.

If a sample point is obtained from sampling through the probability density function. The gradient of the non-zero probability density will point to the largest increased direction of the density. Thus, the samples are more likely to be distributed more along the gradient direction of the probability density.

In the mean shift method, final clustering is affected by two factors, namely, bandwidth of the neighborhood and bandwidth of the color pixel. The following rules are defined for the *x_i_* points that fall in the area of *S_h_*.

In the comparison of the colors of pixels *x* and *x_i_*, the probability density is high when the pixel bandwidth is small. When comparing the distances of pixels *x* and *x_i_*, the short distance bandwidth between *x* and *x_i_* indicates high probability density. Thus, probability density is the product of these two rules.

By substituting the kernel function in Equation (1), we can transform the result of Equation (2) into Equation (4):

(4) $$K_{h_{s}^{2}}{,}_{h_{r}^{2}}(x)=\frac{C}{h_{s}^{2}h_{r}^{2}}K(||\frac{x^{s}-x_{i}^{s}}{h_{s}}|{|}^{2})K(||\frac{x^{r}-x_{i}^{r}}{h_{r}}|{|}^{2})$$

where K(⋅) is the kernel function to solve high dimension disaster. $h_{s}$ represents the distance bandwidth. *h_r_* reflects the color bandwidth:

$$K(||\left(x^{s}-x_{i}^{s}\right)/h_{s}|{|}^{2})$$

indicates the space location of the information:

$$K(||(x^{r}-x_{i}^{r})/h_{r}|{|}^{2})$$

shows the color information. $C/(h_{s}^{2}h_{r}^{2})$ denotes unit density.

### 2.4. Watershed Transformation

Watershed transformation was originally proposed by Beucher *et al.* [29] and improved via rapid implementation methods established by Vincent and Soille [30]. This transformation is traditionally classified as a region-based segmentation approach. Arslan *et al.* [11] also used marker functions to improve the performance of watershed transformation.

In geography, a watershed is a ridge, and a catchment basin is located in the areas on both sides of the ridge with different types of water flow. In gray-scale image processing, watershed transform is applied with the local extremum region as the catchment basin and the boundary of the basin as the ridge line to perform segmentation in gray-scale images. Oversegmentation because of more local extremum region is a well-known limitation of watershed transformation. However, this problem does not significantly influence the proposed WBC adhesion segmentation scheme because precise nucleus groups are regarded as the local extremum region.

## 3. Scheme and Methods

This paper presents novel insights into WBC segmentation by obtaining cell seeds and separating adhesive cells in peripheral blood and bone marrow images under different lighting conditions. The developed algorithm is mainly divided into four phases. The first phase aims to obtain the nucleus and inside seeds from the rg and HSI color spaces. The second phase focuses on obtaining WBCs as outside seeds through mean shift clustering operations, extraction of the C channel component in CMYK model, illumination intensity adjustment, and image enhancement techniques. The third phase intends to solve cell adhesion by using NMWO. The last phase involves post-processing techniques to precisely obtain the nucleus and WBCs. A morphological operation is applied in all phases to improve segmentation accuracy.

In contrast to traditional algorithms [11,14,31], the proposed method is suitable for bone marrow and peripheral blood image, and employs local region color information clustering, illumination intensity adjustment, extraction of the C channel component in the CMYK model, and adaptive threshold techniques, The proposed method exhibits robustness for segmentation of various types of WBCs, even under conditions with similar cytoplasm and background. This method also solves the cell adhesion problem with satisfactory performance.

**Figure 2:**
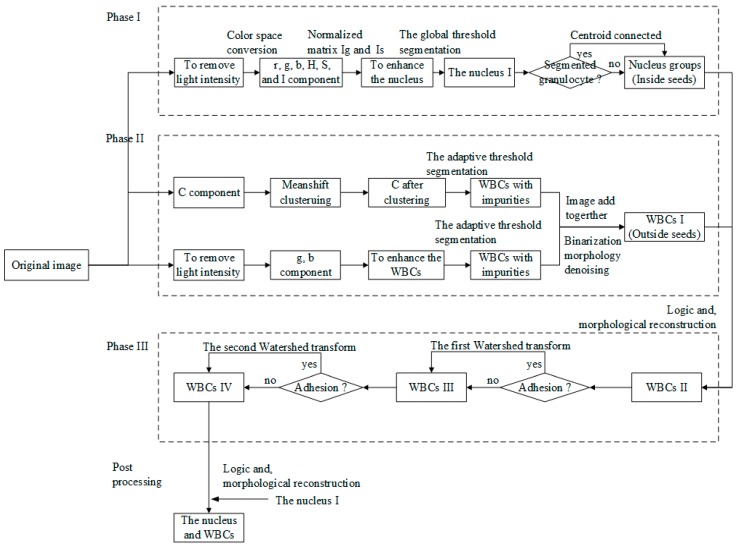
Framework of WBC segmentation.

Figure 2 shows the proposed framework of the segmentation scheme. As shown in the block diagram, the system has four main phases, the details of which are explained in the following subsections.

### 3.1. Morphology Operation

Reconstruction operation is a morphological transformation method that requires a marker binary image, a mask binary image, and a structural element. The marker image should be a subset of the mask image.

In morphological reconstruction, the marker binary image denotes the beginning of transformation, the mask binary image constraints the transformation process, and the structural element defines the connectivity. The refactoring mask binary image from the marker binary image is defined by an iterative process. Refactoring can restore the shape of the image. The accuracy of refactoring depends on the shape and the similarity among structural elements.

In morphology denoising, common algorithms used to extract connected regions are conducted at four connected regions. Area less than *S_1_* are removed in the four connected regions of the nucleus binary image, whereas areas less than *S_2_* are removed in the four connected regions of the individual WBC binary image. In this study, this method is referred to as morphology denoising.

During morphological erosion and dilation, the former removes small isolated features; breaks apart thin, adjoining regions in a feature; and reduces the size of solid objects by “eroding” them at the boundaries. Dilation joins the broken lines to form a contour, which delineates the region of interest.

### 3.2. Phase I

This phase mainly aims to obtain nucleus in a peripheral blood image and nucleus groups to locate WBCs.

Inside seeds, which are also called nucleus groups, can be used to determine the correct number of WBCs and applied as the local extremum region in the process of watershed transformation to avoid oversegmentation. Therefore, accurate acquisition of inside seeds is crucial.

When color information is considered, the nucleus in the g component of the rg chroma space presents lower pixel values than the other components. In the HSI color space, the nucleus in the S component demonstrates higher pixel values than the other components.

**Figure 3:**
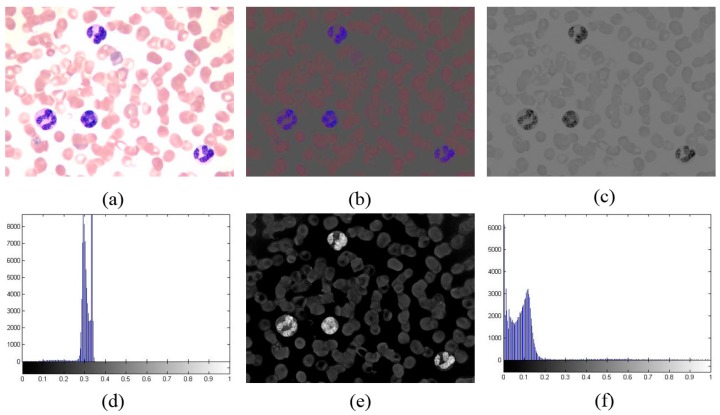
(**a**) Original RGB image in peripheral blood smear; (**b**) Rg chroma image; (**c**) Normalized matrix *I_g_*; (**d**) Histogram of *I_g_*; (**e**) Normalized matrix *I_s_*; (**f**) Histogram of *I_s_*.

Let I be the rg chroma image by Equation (1). The normalized matrices *I_g_* and *I_s_* are denoted by the separated g component in the rg chroma spaces and the S component in the HSI color spaces, respectively. The ranges of g and S can be normalized by:

(5) $$\left\{ \begin{array}{l} I_{g}=(g-g_{m})*255./(g_{M}-g_{m})\\ I_{s}=(S-S_{m})*255./(S_{M}-S_{m}) \end{array}\right.$$

where $g_{M}$ , $g_{m}$ , $S_{M}$ , and $S_{m}$ are the maximum value of g , the minimum value of g , the maximum value of S, and the minimum value of S, respectively. Figure 3 shows the normalized matrices *I_g_* and *I_s_*, as well as their histogram.

The enhanced image (*I_E_*) can be expressed as follows:

(6) $$I_{E}=\left\{ \begin{array}{l} I_{s}./I_{g}\text{ if }I_{g}>0\\ \text{0 otherwise } \end{array}\right.$$

The histogram in Figure 3 shows that the nucleus contains a small gray level in $I_{g}$ and a large gray level in $I_{s}$ , whereas the background and RBCs present low gray levels in $I_{s}$ and high gray levels in $I_{g}$ . Figure 3 shows that $(I_{s\_nucleus}./I_{g\_nucleus})\gg 1$ , $(I_{s\_rbc}./I_{g\_rbc})<1$ , and $(I_{s\_background}./I_{g\_background})<1$ . where $I_{s\_nucleus}$ , $I_{g\_nucleus}$ , $I_{s\_rbc}$ , $I_{g\_rbc}$ , $I_{s\_background}$ , and $I_{g\_background}$ are the gray levels of nucleus in $I_{s}$ , nucleus in $I_{g}$ , RBCs in $I_{s}$ , RBCs in $I_{g}$ , background in $I_{s}$ , and background in $I_{g}$ , respectively.

Adaptive global threshold segmentation proposed by Otsu is applied for $I_{E}$ to obtain the inside seed binary figure. The mathematical morphology method is used to eliminate platelets or other impurities in the binary figure. The nucleus in a peripheral blood image can also be obtained through the same method, which produced accurate results in [8]. For parts of the image of bone marrow blood cells, the difference between the tint of the cytoplasm and the nucleus is too small to be precisely distinguished. This method can also be used to segment WBCs in several AML blood images. Regarding texture, the method can also produce good recognition results in an automatic recognition system.

Nucleus groups are difficult to obtain because of segmented granulocytes with multiple cores. A centroid-connected operation is proposed in this study to convert multiple cores into a single core. The area and distance between multiple cores have a certain range. We assume the existence of two cores, $a_{1}$ and $a_{2}$ , where $s_{1}$ and $s_{2}$ are the areas of $a_{1}$ and $a_{2}$ , respectively, and $l_{1}$ is the minimum distance between $a_{1}$ and $a_{2}$ . If $s_{11}$ < $s_{1}$ < $s_{12}$ , $s_{11}$ < $s_{2}$ < $s_{12}$ , and $l_{1}$ < $l_{11}$ , then multiple cores exist in the inside seed binary image. In addition, $s_{11}$ , $s_{12}$ , and $l_{1}$ are the thresholds determined by the area range of a single core and the general spacing of multiple cores. We assume that $\left(x_{1},y_{1}\right)$ and $\left(x_{2},y_{2}\right)$ are the centroid coordinates of $a_{1}$ and $a_{2}$ , respectively. The two coordinates are adopted to perform centroid-connected operation and obtain the following nucleus groups:

(7) $$\left\{ \begin{array}{l} A=\max(x_{11}-T_{2},0)\\ B=\min(x_{22}+T_{2},xx)\\ C=\max(y_{11}-T_{2},0)\\ D=\min(y_{22}+T_{2},yy) \end{array}\right.$$

**Figure 4:**
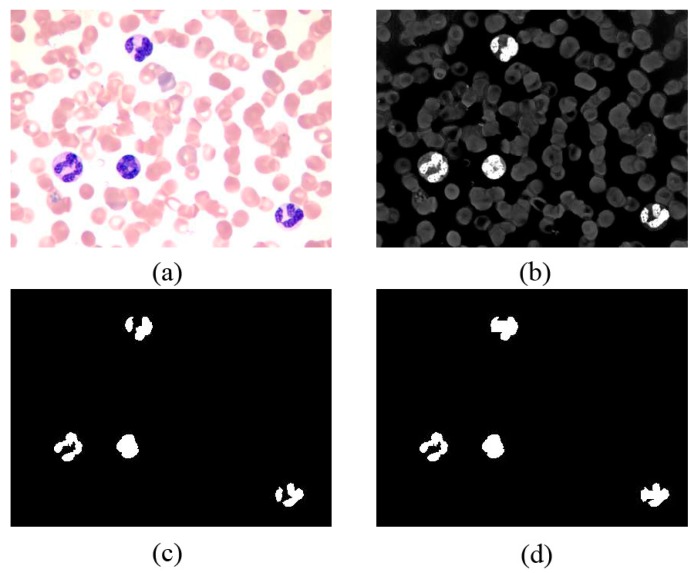
(**a**) Original RGB image in peripheral blood smear; (**b**) Enhanced nucleus images; (**c**) Segmented nucleus binary image; (**d**) Inside seeds after the centroid-connected operation.

In Equation (7), $T_{2}$ is the fluctuation range of the centroid. $x_{11}$ , $x_{22}$ , $y_{11}$ , and $y_{22}$ are the minimum value of $\left\{x_{1},x_{2}\right\}$ , the maximum value of $\left\{x_{1},x_{2}\right\}$ , the minimum value of $\left\{y_{1},y_{2}\right\}$ , and the maximum value of $\left\{y_{1},y_{2}\right\}$ , respectively. 0, xx , 0, and yy are the minimum value of the X-axis, the maximum value of the X-axis, the minimum value of the Y-axis, and the maximum value of the Y-axis, respectively. Figure 4 presents the process of obtaining inside seeds.

We obtain the maximum and minimum values to prevent the boundaries of the centroid from exceeding the range of the axes. A rectangular shape is constructed using the four vertices: (A,C) , (A,D) , (B,C) , and (B,D) . Within this rectangular shape, the pixel value is 1, and the value of the others pixels is 0. Finally, the multiple cores are connected as shown in Figure 4c,d. 

### 3.3. Phase II

The outside seed is a binary image that contains WBCs and certain impurities. The outside seed plays an important function in segmentation of adhesive cells and obtains WBCs accurately. In this phase, two strategies are used to obtain WBCs, namely, mean shift clustering and WBC enhancement. The combination of these two strategies confers two advantages for obtaining outside seeds. The operations strengthen the weak areas of WBCs and overcome the lighting effects on image quality to some extent.

#### 3.3.1. Mean Shift Clustering

**Figure 5:**
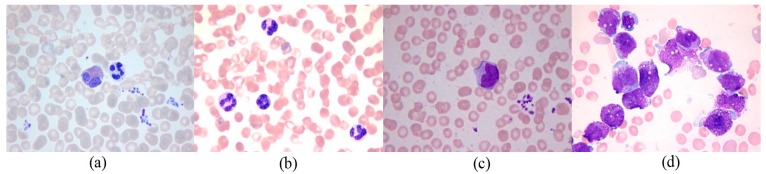
(**a**) Original RGB image for WBCs with 1 normal eosinophil, 1 normal staff neutrophil; (**b**) Original RGB image for WBCs with 4 normal neutrophils; (**c**) Original RGB image for WBCs with 1 normal monocyte; (**d**) Original RGB image for AML WBCs.

**Figure 6:**
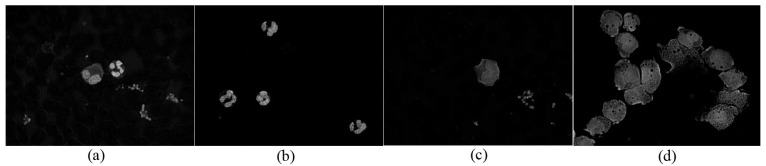
C component in the CMYK color space of four images in Figure 5.

**Figure 7:**
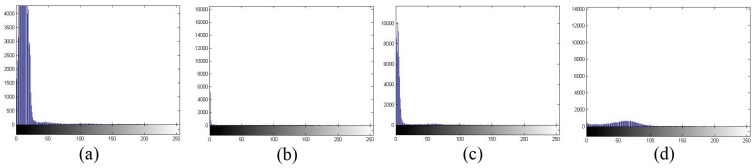
Histogram of the C component of four images in Figure 5.

Mean shift clustering is an algorithm used to smooth image based on color and distance in image segmentation. The mean shift procedure is applied to cluster data points, whose trajectories of the gradient ascent lead to the same mode. Figure 5 shows the original RGB image of four types of WBCs. Figure 6 and Figure 7 show the C component and the histogram of C the component in the CMYK color space, respectively. Selection of the C component to obtain WBCs in the CMYK color space is a suitable technique for mean shift clustering based on color and space for two reasons. First, the brightness of the nucleus is stronger than that of the cytoplasm, whereas the brightness of the cytoplasm is stronger than that of RBCs and the background. Second, cyan is a mixture of green and blue. The cyan domain, which is relatively small in the visible spectrum at wavelengths of 480 nm to 510 nm, presents identical color brightness. By placing the color image in a 3D feature space, where one component represents the C component and two other components represent the *x* and *y* spatial coordinates of the pixel, the image can be segmented through 3D mean shift clustering. This technique is highly effective for clustering statistics iteration. For clustering, the visual image derived from the segmentation method is combined with the color and space information.

**Figure 8:**
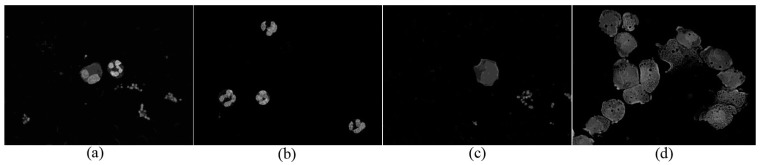
C component after clustering of four images in Figure 5.

The simplest uniform distribution is the kernel function that used in this study. After clustering through selection of the appropriate kernel distance bandwidth ( $h_{s}$ ) and color bandwidth ( $h_{r}$ ), the C component image is shown in Figure 8.

As shown in Table 1, the width range of the WBCs is 26 to 74. Generally, the width of most WBCs is within the mid-value of 50. In the mean shift clustering algorithm, iterations are conducted four times by default to determine a mode in one square with a side length of (2 $h_{s}$ + 1). In theory, $h_{s}$ = 3 or $h_{s}$ = 5 is a suitable kernel distance bandwidth.

**Figure 9:**
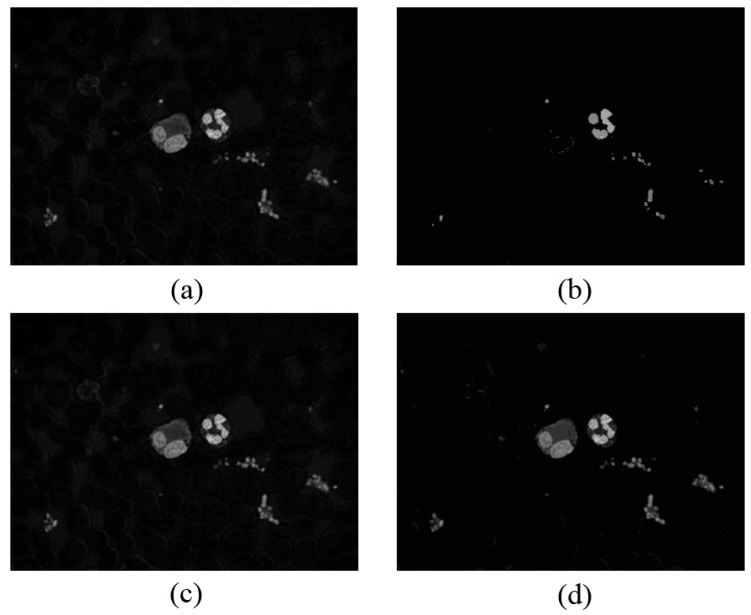
(**a**) Original C component image; (**b**) Segmented with ( $h_{s}$ , $h_{r}$ ) = (1,13); (**c**) Segmented with ( $h_{s}$ , $h_{r}$ ) = (13,1); (**d**) Segmented with ( $h_{s}$ , $h_{r}$ ) = (3,3).

Figure 6 and Figure 7 show that the RBCs and background exhibit smaller intensity values than the nucleus and cytoplasm, with $h_{r}$ = 3 as the color bandwidth. Figure 9 shows the clustering results with different bandwidths. As shown in Table 1 and Table 2, we assume that the area of WBCs is N times higher than the area of the nucleus. After mean shift clustering, we set the segmentation threshold $T_{3}$ as follows to convert the image intensity in 0–1:

(8) $$\left\{ \begin{array}{l} S_{red\;b}=\frac{N*S_{nucleus}}{S_{image}}\\ gray_{Sum\_c}=\sum_{i=0}^{T_{g}}gray_{C}(i)\\ T_{3}=T_{g}\text{ if }S_{red\;b}=gray_{Sum\_c} \end{array}\right.$$

where $S_{nucleus}$ is the area of nucleus, and $S_{image}$ is the area of the whole image. $gray_{C}(i)$ shows the gray value frequency in the C component, $gray_{Sum\_c}$ is the summation of gray value frequency, and i is the gray value from 0 to $T_{g}$ . (peripheral blood images, *N* = 3; bone marrow images, *N* = 1.5).

**Table 1:** Characteristic parameters for seven types of WBCs.

Cell Types	Area	Height	Weight	Roundness
segmented neutrophil	708~1797	28~53	30~48	1.49~2.31
staff neutrophil	939~2236	32~58	37~59	1.57~2.54
lymphocyte	**460**~2468	**26**~65	27~61	1.52~2.34
monocyte	1777~3367	49~70	47~**74**	1.51~2.48
eosinophil	942~2216	30~55	32~55	1.67~3.09
basophil	943~1932	36~63	31~51	1.51~3.08
AML WBCs	553~3041	27~60	24~67	1.47~3.03

**Table 2:** Characteristic parameters of the nucleus.

Cell types	Area	Height	Weight	Distance between Two Cores
nucleus of segmented neutrophil	**124**~**872**	**10**~39	**10**~39	**0**~ $\sqrt{41}$
nucleus of staff neutrophil	418~1029	12~52	13~50	
nucleus of lymphocyte	437~1018	23~44	23~39	
nucleus of monocyte	970~1640	33~61	32~53	
nucleus of eosinophil	426~2157	31~53	32~53	
nucleus of basophil	858~1716	31~47	30~50	

**Figure 10:**
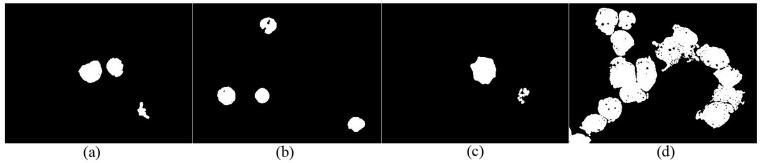
WBC segmentation results of four images in Figure 5.

From the adaptive threshold segmentation for the C component after clustering, we obtain WBCs, as shown in Figure 10.

#### 3.3.2. WBC Enhancement

**Figure 11:**
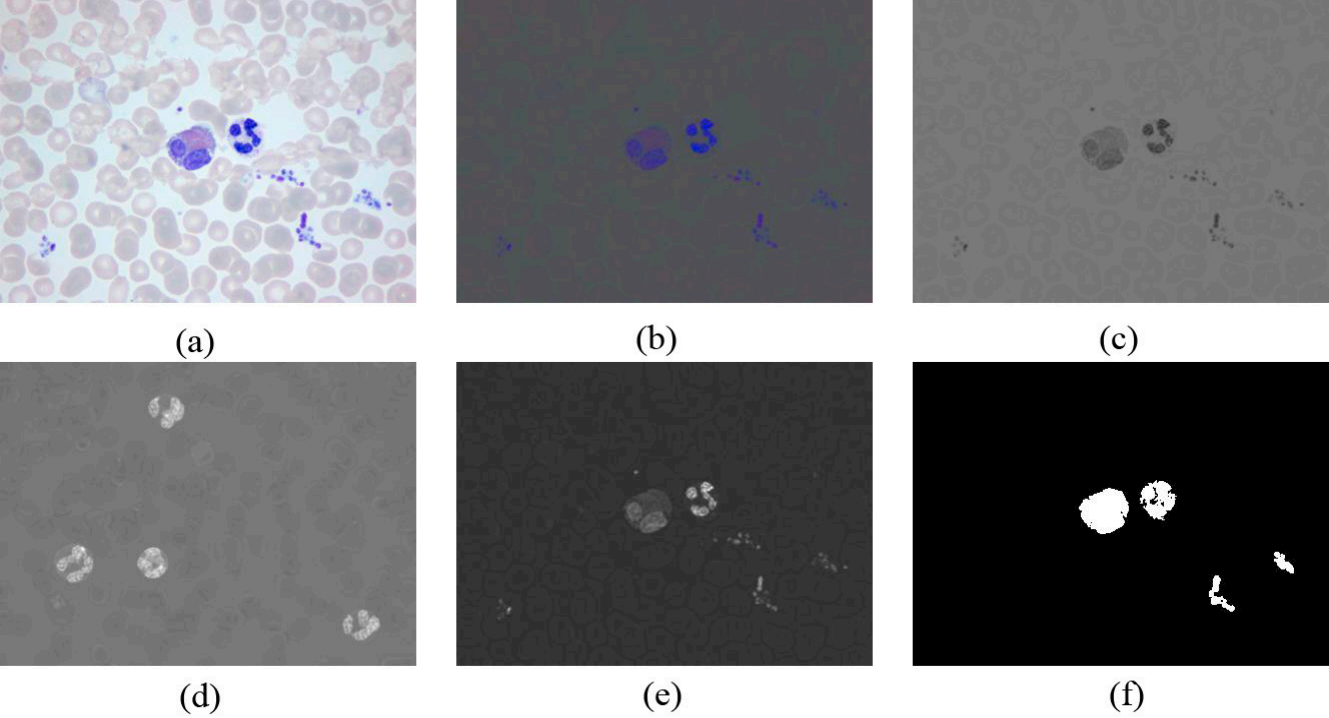
(**a**) Original RGB image in peripheral blood smear; (**b**) Rg chroma image; (**c**) The g component of rg chroma image; (**d**) The b component of rg chroma image; (**e**) Enhanced image ( $I_{en}$ ); (**f**) Segmentation result.

When the components of the rg chroma image are considered, WBCs in the g component present smaller pixel values (Figure 11a) than the other components, whereas WBCs in the b component (Figure 11b) demonstrate pixel values than the other components. After removing the light intensity, the enhanced image $I_{en}$ can be defined by:

(9) $$I_{en}=\left\{ \begin{array}{l} b./g\text{ if }g(i,j)>\text{0}\\ \text{0 otherwise} \end{array}\right.$$

Figure 11c shows the enhanced image. Adaptive threshold segmentation for the enhanced image can be used to obtain WBCs with some impurities, as shown in Figure 11d.

The adaptive segmentation threshold $T_{4}$ is set as follows:

(10) $$\left\{ \begin{array}{l} S_{red\;b}=\frac{N*S_{nucleus}}{S_{image}}\\ gray_{Sum\_en}=\sum_{i=0}^{T_{g2}}gray_{en}(i)\\ T_{4}=T_{g2}\text{, if }S_{red\;b}=gray_{Sum\_en} \end{array}\right.$$

where $S_{nucleus}$ is the area of the nucleus, $S_{image}$ is the area of the entire image, $S_{red\;b}$ is the area of RBCs and the background, $gray_{en}(i)$ is the gray value frequency of the enhanced image, $gray_{Sum\_en}$ is the summation of the gray value frequencies and *i* is the gray value from 0 to $T_{g2}$ .

#### 3.3.3. Obtaining the WBC Region

**Figure 12:**
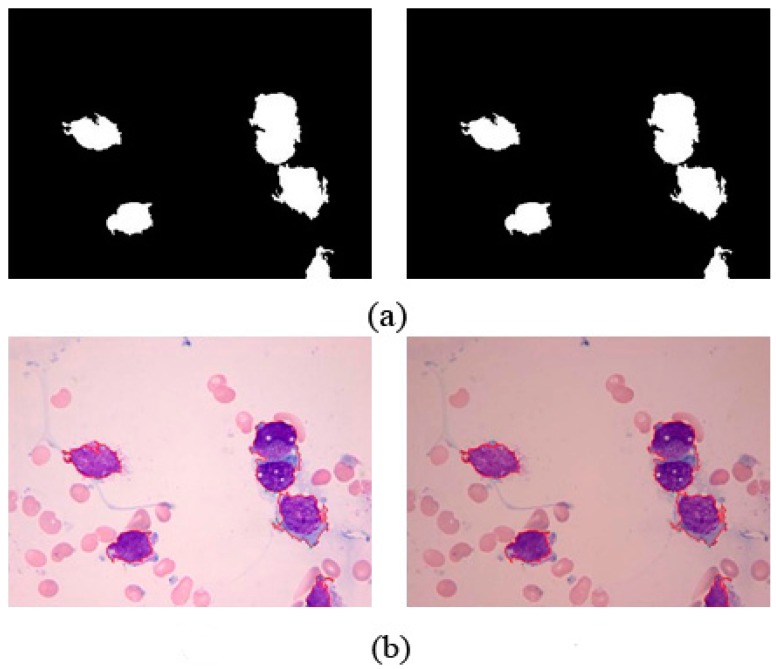
(**a**) Binary image of WBC segmentation in Figure 1a; (**b**) Segmentation results for Figure 1a under different light conditions (segmented WBCs marked with red curves).

**Figure 13:**
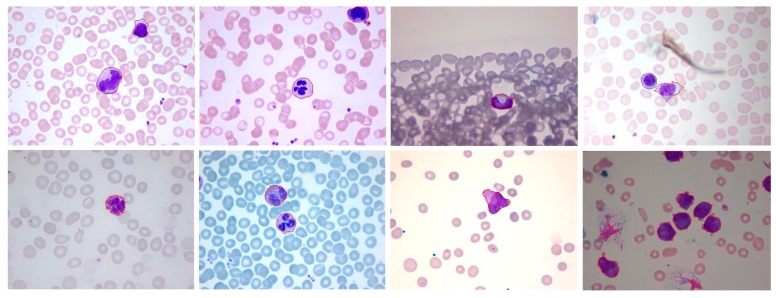
Segmented results for WBCs with 1 monocyte, 1 staff neutrophil, 2 segmented neutrophils, 1 basophil, 1 eosinophil, 4 lymphocytes, and 9 AML cells (segmented WBCs are marked with red curves).

Two WBC binary images segmented by mean shift clustering operations and WBC enhancement operations are add before binarization and morphology denoising. The required WBC binary images are determined and presented in Figure 12 and Figure 13.

### 3.4. Phase III

Watershed segmentation is a mathematical method based on the theory of topology for morphology segmentation. NMWO is a watershed algorithm marked by the nucleus. If we assume that one WBC has one nucleus and the nucleus is not an adhesive type, the nucleus can be regarded as a local extremum region to mark WBCs. The watershed ridge line of the local extremum region can serve as the boundary of WBCs to separate adhesive WBCs. When the nucleus is adhesive or WBCs and RBCs are of the adhesive type, a second watershed transformation process is required to separate adhesive cells.

If we assume the presence of target $a_{i}$ , then r denotes the roundness value and $s_{3}$ shows the area of target $a_{i}$ . If r < R and $s_{3}$ > $S_{3}$ , then the target is the adhesive cells in this study. R and $S_{3}$ are the roundness and area thresholds, respectively. The overall description of the NMWO algorithm is described as follows:

Step 1:  Obtain and modify inside seeds and outside seeds by using the mean shift algorithm and the morphology operation.
Step 2:  Determine whether cell adhesion occurs or not in WBCsII . If yes, proceed to the following steps; if no, end.
Step 3:  Generate the map of distances, named Im , from the black pixel to the white pixels of the inside seeds.
Step 4:  Apply the watershed algorithm to Im . The watershed ridge line shown in WBCsII can be used to obtain separating blood cell images ( WBCsIII ).
Step 5:  Determine whether cell adhesion occurs or not in WBCsIII . If yes, do the following steps; if no, end.
Step 6:  Obtain the local extremum region $b_{i}$ [21] on the adhesion target $a_{i}$ individually. The local extremum $R_{s}$ is designed by cell size. Perform adaptive iteration corrosion on adhesion target $b_{i}$ individually until the number of targets increases or does not merely disappear.
Step 7:  Apply the watershed algorithm to $b_{i}$ one by one to obtain the watershed ridge lines. The watershed ridge line displayed in WBCsIII can obtain the separating blood cell images ( WBCsIV ).
Step 8:  End.

**Figure 14:**
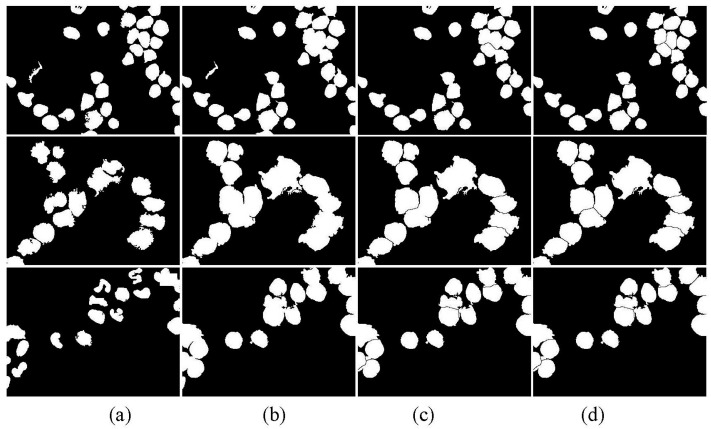
(**a**) Inside seeds; (**b**) Outside seeds; (**c**) First watershed transformation result; (**d**) Second watershed transformation result.

Figure 14b shows the preliminary binary image of WBCs. Most WBCs are adhesive. As shown in Figure 14d, all adhesive cells are separated after applying the NMWO segmentation algorithm. Multiple sets of successful segmentation results show that the adhesion segmentation method is highly effective.

### 3.5. Post-Processing

Post-processing is required to accurately obtain nucleus and WBCs. Applying “logic and” between the second watershed transformation binary figure ( WBCsIV ) and the nucleus can address nucleus adhesion. When WBCsIV is a mask and the nucleus is a marker binary image, morphological reconstruction for WBCsIV and nucleus can remove impurities that adhered in WBCsIV . The final result shows the separation of WBCs in the binary image.

## 4. Experimental Results

### 4.1. Data Set

Two sets of blood smear images were used in this study. Dataset 1 was collected from the Second Affiliated Hospital of Shandong University. Blood smears were created by the Wright staining method. A ternary Olympus microscope with an oil lens of 100× magnification and CCD was used to collect data images. The 24-bit RGB images were obtained under different lighting conditions for three types of smears (normal peripheral blood, M3 bone marrow, and M5 bone marrow) sampled from more than 10 individuals. A total of 306 images with 774 WBCs were captured with a resolution of 2080 × 1542 pixels. In dataset 2, 108 images were downloaded from the ALL-IDB1 dataset [32]. These JPEG images were captured in the RGB format with three resolutions of 2592 × 1944 pixels, 1712 × 1368 pixels, and 1226 × 652 pixels.

### 4.2. Morphology Preference

Before the image processing, the original image was resized to 0.2 times higher to improve the efficiency of segmentation system.

Most WBCs are round and convex in shape, and the roundness and area of individual cells present a certain range. However, the shapes and roundness appear checkered and non-convex when multiple cells form larger regional areas or myeloplast lesions. After repairing the binary image of WBCs by morphological operations, chain code tracking is used to obtain the morphological characteristic parameters of WBCs.

The main morphological characteristic parameters are area, perimeter, length, width, and roundness. We divided WBCs into seven cell types, namely, segmented neutrophil, staff neutrophil, lymphocyte, monocyte, eosinophil, basophil, and AML WBCs. More than 30 WBCs are involved in the parameter statistics for every cell type.

The main morphological characteristic parameters of WBCs and the nucleus in dataset 1 are shown in Table 1 and Table 2, respectively. In this study, most parameters were designed based on the morphological characteristic parameters in Table 1 and Table 2:

(1)
$S_{1}$ and $S_{2}$ . As shown in Table 1, the minimum area in the nucleus is 124 in segmented neutrophils, and the minimum area in WBCs is 460. Thus, we select $S_{1}$ = 100 and $S_{2}$ = 350 as the area thresholds to eliminate platelets and noise in morphological denoising.
(2)
$T_{2}$ : Fluctuation range of the centroid, which may not be located in the cores. We set a fluctuation range around the centroid to connect multiple cores into one nucleus and comprehensively consider the length and width of WBCs, as well as the distances of the multiple cores. $T_{2}$ = 5 is a suitable fluctuation range.
(3)
$l_{11}$ : The minimum distance threshold of multiple cores. We calculated the range of distances between multiple cores, which is from 0 to $\sqrt{41}$ . $l_{11}$ = $\sqrt{55}$ is a suitable distance threshold.
(4)
$s_{11}$ and $s_{12}$ : Area thresholds of one core. The range of the area is 124 to 872 for one core. The area whose value is within the range of 100 to 950 can be one of the judgment conditions to distinguish segmental neutrophils. Thus, $s_{11}$ = 100 and $s_{12}$ = 950.
(5)
$S_{3}$ and R . $S_{3}$ and R are the area and roundness threshold of the adhesion target, respectively. In most cases, the area of the adhesive target is higher than 3500, whereas the roundness of the adhesive target is higher than 3.00. Thus, $S_{3}$ = 3500 and R = 3.00.
(6)
$R_{s}$ . $R_{s}$ is the local extremum in the second watershed transformation. $R_{s}$ is designed based on WBC size. The minimum length ml of WBCs is 24 in terms of AML WBCs shown in Table 1. $R_{s}$ is a little less than ml/2 , Thus, we selected $R_{s}=7$ as a suitable local extremum.

The morphological characteristic parameters of WBCs and the nucleus are modified with the image resolution pixels. We also calculate the morphological characteristic parameters of dataset 2 by using the similar method to that used in dataset 1.

### 4.3. Segmentation Evaluation

Identification of bone marrow leukocytes can provide a preliminary and auxiliary diagnosis of leukemia. To date, the recognition rate of bone marrow cells by several senior leukemia experts is only 60% to 70%. Degenerative bone marrow cells and other cell types affect the distinction of leukemia in a computer recognition system. Therefore, cells with characteristics similar to WBCs, such as degenerative bone marrow cells and metarubricytes, were regarded as WBCs in this study.

A total of 414 images, 952 individual WBCs, and 598 adhesive WBCs were used from the dataset to complete the segmentation experiments. The dataset contained 260 peripheral blood WBCs, which consisted of 45 basophils, 31 eosinophils, 88 neutrophils, 46 lymphocytes, and 50 monocytes in the peripheral blood images, as well as 514 AML WBCs, and approximately 776 ALL-IDB1 WBCs in the bone marrow images. We compared our algorithm with three methods, namely, color-based clustering [14], color and shape transformation [11], and simple linear iterative clustering (SLIC) [31].

**Figure 15:**
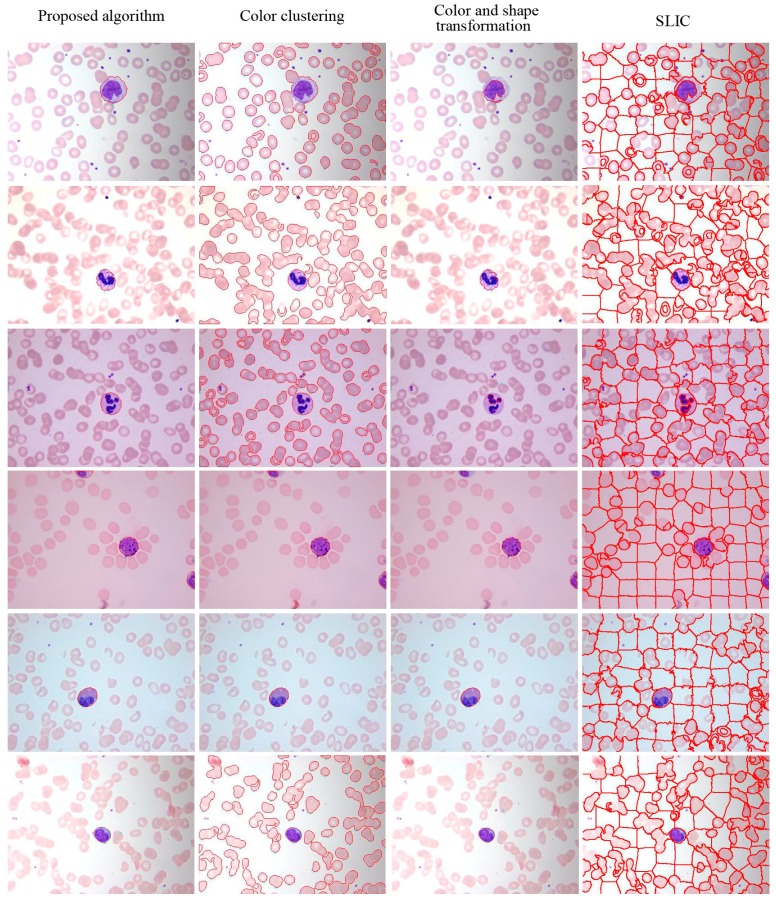
Peripheral blood images of (top to bottom) monocyte, staff neutrophil, segmented neutrophil, basophil, eosinophil, and lymphocyte (segmented WBCs marked with red curves).

**Figure 16:**
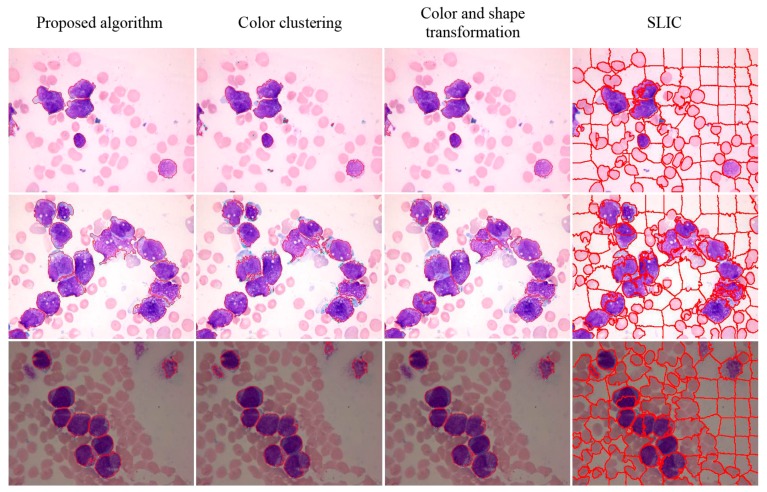
Segmentation results of AML images (segmented WBCs marked with red curves).

**Figure 17:**
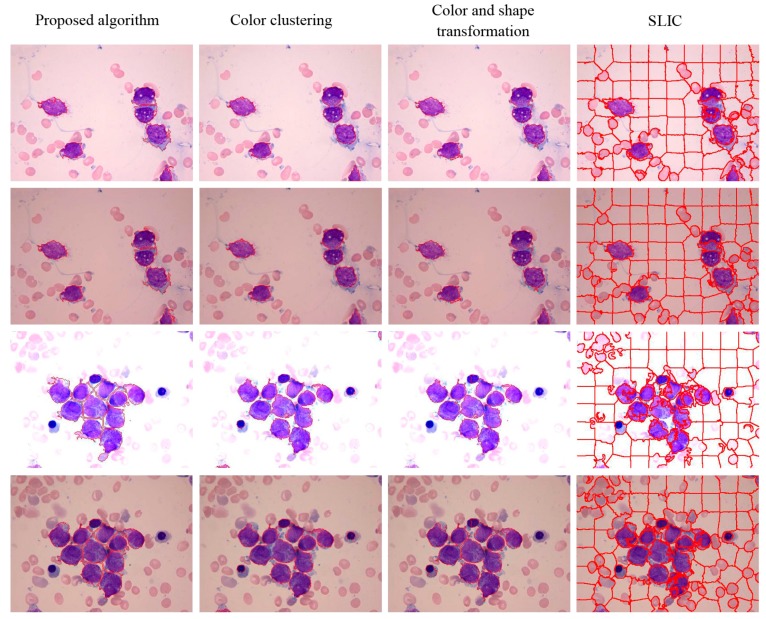
Two sets of Images under different light conditions (segmented WBCs marked with red curves).

**Figure 18:**
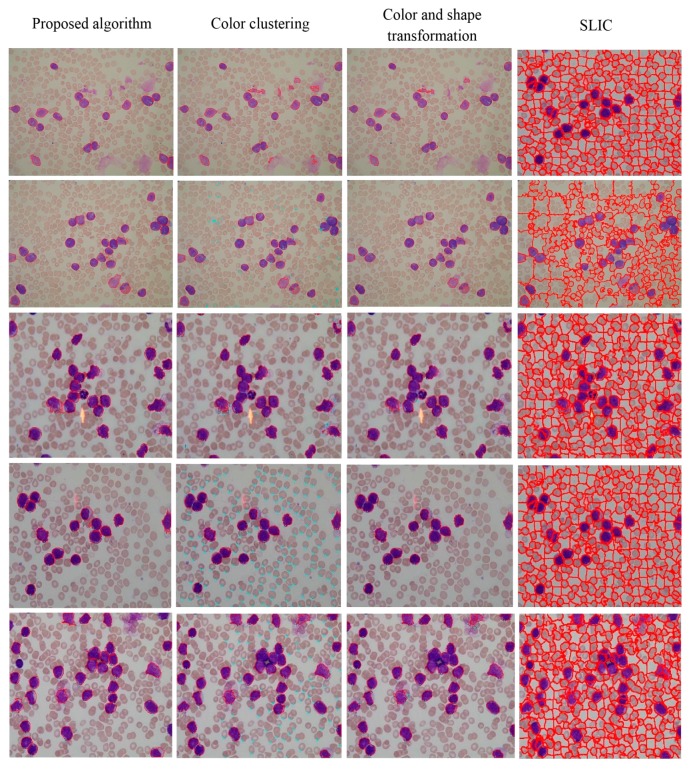
Segmentation results of images with disease in the ALL-IDB1 dataset (segmented WBCs marked with red curves).

**Figure 19:**
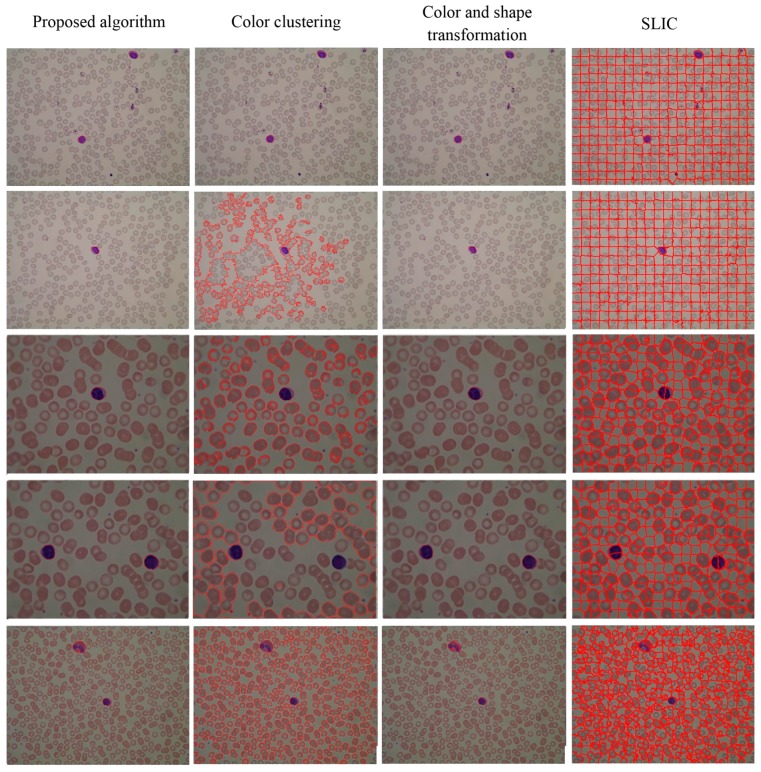
Segmentation results of normal images in the ALL-IDB1 dataset (segmented WBCs marked with red curves).

The visual results for WBC segmentation are presented in Figure 15, Figure 16, Figure 17, Figure 18 and Figure 19. These resulting measures are also consistent with the quantitative results presented in Table 3, Table 4 and Table 5.

**Table 3:** Quantitative comparison of four algorithms.

	TP	OVER SEGM	UNDERSEGM	FP	FN
Proposed algorithm	**1481**	19	**44**	**10**	**69**
Color clustering	1332	**9**	511	>800	218
Color and shape transformation	1407	25	73	35	143
SLIC	1327	286	57	>100	223

**Table 4:** Quantitative comparison of four algorithms by using F-measure.

	Proposed Algorithm	Color Clustering	Color and Shape Transformation	SLIC
P	**99%**	<62.5%	97.6%	<93%
R	**95.5%**	86%	90.8%	85.6%
F1	**97%**	<72.4%	94.1%	<89.1%

**Table 5:** Quantitative comparison in different cell types in two datasets.

		Proposed Algorithm	Color Clustering	Color and Shape Transformation	SLIC
Dataset 1	Lymphocyte	**43**	33	39	38
	Monocyte	**49**	35	45	40
	Eosinophil	29	22	**30**	23
	Basophil	**44**	33	42	35
	Neutrophil	**83**	57	61	49
	AML WBCs	**472**	301	435	368
Dataset 2	ALL-IDB1 WBCs	**698**	331	657	431

In [14], the image is transformed into the Lab color space before image pixels are clustered by running K-means on the a and b channels. The image pixels, such as a and b channels, are plotted in the feature space and clustered into three groups based on their features. The cluster that contains circular objects was selected to represent WBCs. Connected objects with small sizes were removed in a post processing step.

Color clustering implemented for cell segmentation performs effectively on bone marrow images because of the color similarity between the cytoplasm and nucleus in these images. However, for a few peripheral blood images, the pixel values of the cytoplasm and nucleus in the *a* or *b* components are easily distinguished, as shown in Figure 15 and Figure 19.

In [11], an intensity map can be obtained by color transformation in the first stage, and the coarse mask can be obtained by applying the Otsu’s method on the intensity map. The boundaries were also refined by applying active contours without edges [33], which produced an active contour by minimizing the energy function on the intensity map. Therefore, the mask produced after active contours application was delimited by the coarse mask, followed by the WBC binary mask. The problem of cell adhesion was solved by watershed transform based on distance transformation [11].

The experimental results of [11] showed that the method is suitable for bone marrow image in which WBCs considerably differ from the background. Two discussion-worthy points can be inferred. First, the intensity map cannot describe the characteristics of all types of WBCs. Second, the active contour model cannot deal with the intensity of inhomogeneous images.

Comparison of NMWO with marker-function watershed transformation [11] showed that the local extremum region can be obtained depending on the shape of the adhesive cells in the binary image. When the adhesive cells appear to be in pieces, an adaptive iteration method combined with NMWO used as the watershed operation can achieve more accurate segmentation results than the marker-function watershed transformation. In this study, the use of the nucleus and adaptive iteration of WBCs as the reception basin for segmentation of adhesive cells in complex bone marrow images achieved more accurate results than other methods.

The brightness of the nucleus is generally higher than that of the cytoplasm, RBCs, and background, in the peripheral blood and bone marrow images. In our experiments, we combined spatial and color similarities to obtain WBCs and achieve high accuracy for every type of WBCs through mean shift clustering. The K-means algorithm is based on color clustering, and differs from mean shift clustering. When different cell types simultaneously appear in an image, the K-means algorithm, in contrast to mean shift clustering, cannot adapt to large changes in the pixel intensity of different types of WBCs. SLIC is another segmentation method that differs from mean shift clustering in terms of color and space. In the preprocessing stage, SLIC is suitable for obtaining image boundaries, but not for WBC segmentation.

In this study, the proposed algorithm based on color features and adaptive thresholds achieved accurate segmentation result for WBC segmentation. Two segmentation strategies were performed to overcome common problems in segmenting weak areas, such as the cytoplasm. The proposed algorithm is considered robust for different types of WBCs under different lighting conditions.

Most of the nucleus did not show adhesion, and one WBC contained one nucleus group. We applied nucleus groups as the local extremum regions, and the watershed transformation based on tag [21] was used for segment-overlapping WBCs. Thus, the complexity of leukocyte adhesion is considerably reduced to a certain extent. When the nucleus showed adhesion, we applied the second watershed transformation based on the adaptive iteration method for overlapping WBCs. The application of NMWO to segment adhesive cells achieved more accurate results than other methods for complex bone marrow images.

The performance of the algorithms was also evaluated based on F-measure [34]. TP shows the number of correct WBC segmentations, FP reflects the number of RBCs and other impurities, and FN denotes the number of leaking and broken WBCs. Table 3 and Table 4 show the comparison of the overall performance in terms of the F-measure of the four algorithms, whereas Table 5 enumerates the correct segmentation of different cell types in the two datasets.

For both datasets, the proposed method exhibit satisfactory performance for WBC segmentation. The experimental results are presented in Table 3, Table 4 and Table 5. Table 3 and Table 4 show that the proposed algorithm achieves better performance than other traditional methods, such as color clustering [14], color and shape transformation [11], and SLIC [31]. Table 5 further demonstrates that the proposed method outperforms other traditional methods for the private and public datasets. Figure 15, Figure 16, Figure 17, Figure 18 and Figure 19 present the visual comparison between our method and other traditional methods, with the proposed method generating more accurate segmentation results.

The proposed method achieves high performance for WBC segmentation in peripheral blood and bone marrow images. Adhesion segmentation accuracy is a second advantage of the proposed method over traditional methods, as shown in Table 3 and Figure 14 and Figure 16, Figure 17 and Figure 18. However, the proposed algorithm has a few disadvantages. For example, the watershed ridge line does not match the boundaries of cells in a few images. If the degree of cell adhesion and morphology in the image is very complex, other algorithms with reasonable and effective morphological characteristics should be used to solve this complicated problem in future work.

## 5. Conclusions

This paper presents novel insights into WBC segmentation by obtaining cell seeds and separating adhesive cells in peripheral blood and bone marrow images under different light conditions. Color space conversion, mean shift clustering, and illumination intensity adjustment were applied to obtain WBCs as outside seeds. Nucleus enhancement and centroid connection were also employed to obtain inside seeds in different color spaces. The morphological and NMWO operations were subsequently conducted in WBC adhesion segmentation. The proposed method presents a reasonable processing time and provides accurate results.

The proposed method exhibits high robustness and satisfactory performance for WBC segmentation in peripheral blood and bone marrow images. Adhesion segmentation accuracy is the second advantage of the proposed method over traditional methods.

Adhesion segmentation for the diagnosis of complicated abnormal cells in bone marrow diseases remains a great challenge. In addition, distinguishing between ALL and AML or identifying M3 and other AML diseases via an automatic identification system, as mandated by the current WHO standard requires further research.
